# Parallel Processing Method for Airborne Laser Scanning Data Using a PC Cluster and a Virtual Grid

**DOI:** 10.3390/s90402555

**Published:** 2009-04-14

**Authors:** Soo Hee Han, Joon Heo, Hong Gyoo Sohn, Kiyun Yu

**Affiliations:** 1 School of Civil and Environmental Engineering, Yonsei University / 134 Sinchon-dong Seodaemun-gu, Seoul 120-749, Korea; E-Mails: scivile@yonsei.ac.kr; sohn1@yonsei.ac.kr; 2 Department of Civil, Urban and GeoSystem Engineering, Seoul National University / 599 Gwanak-ro, Gwanak-gu, Seoul 151-742, Korea; E-Mail: kiyun@snu.ac.kr

**Keywords:** ALS, LiDAR, Parallel processing, Virtual grid, PC cluster, DSM, DTM

## Abstract

In this study, a parallel processing method using a PC cluster and a virtual grid is proposed for the fast processing of enormous amounts of airborne laser scanning (ALS) data. The method creates a raster digital surface model (DSM) by interpolating point data with inverse distance weighting (IDW), and produces a digital terrain model (DTM) by local minimum filtering of the DSM. To make a consistent comparison of performance between sequential and parallel processing approaches, the means of dealing with boundary data and of selecting interpolation centers were controlled for each processing node in parallel approach. To test the speedup, efficiency and linearity of the proposed algorithm, actual ALS data up to 134 million points were processed with a PC cluster consisting of one master node and eight slave nodes. The results showed that parallel processing provides better performance when the computational overhead, the number of processors, and the data size become large. It was verified that the proposed algorithm is a linear time operation and that the products obtained by parallel processing are identical to those produced by sequential processing.

## 1. Introduction

The construction and updating of 3D spatial databases for urban areas by an airborne laser scanner (ALS) has grown in popularity [1–2]. However, the enhancement of the scanning devices and the increasing size of coverage areas has created large volumes of scanned data, necessitating the development of efficient ALS-data-processing technologies. Shan and Sampath [3] rapidly separated ground from non-ground features with one-dimensional filtering between two consecutive points along scan-lines of raw ALS data. Han *et al.* [4] directly classified raw ALS data into homogeneous groups by an efficient method that utilizes scan-line characteristics. Among the products generated from ALS data, a raster digital surface model (DSM) and digital terrain model (DTM), respectively, can be extensively utilized by various GIS applications. ALS technology’s direct, swift and accurate surveying of ground with enhanced point density makes it ideal for DSM and DTM generation. However, the sharply increased, up-to-terabyte-level data quantities that result, represent a serious data processing problem. As data sizes and the complexity of analyzing methods in GIS and remote sensing have grown, parallel processing has been highlighted as a solution [5–8]. Parallel processing, though a potential ALS-data-processing solution, has not been actively employed in the field. Furthermore, because traditional algorithms might not run effectively in a parallel environment, their modification to a parallel structure is first necessary if parallel processing is to be most effectively utilized. Another problem is that point searches of particular locations cannot be completed in a constant time if the scanned points are not arranged on a proper data structure, because, unlike raster images, they are irregularly distributed geometrically. Thus, the specification of an appropriate data structure and a proper data processing methodology are both necessary if the intended efficiency in processing enormous amounts of ALS data is to be realized.

This paper proposes, as a new framework for the efficient processing of enormous amounts of ALS data, a parallel processing method using a PC cluster and a virtual grid. To test the applicability of the method, a raster DSM was generated from raw ALS point data by interpolating with inverse distance weighting (IDW), and a raster DTM was produced from the DSM by local minimum filtering. A methodology of dealing with boundary data and of selecting interpolation centers in the parallel processing was designed to ensure the same result from the sequential processing. In the present study, results of sequential processing were compared with those of parallel processing. Some standards for assessing parallel processing algorithms were adopted for the purpose of evaluating the computational performance of the proposed algorithm.

## 2. Background

### 2.1. ALS Data Structure and Virtual Grid

ALS data consists of points distributed irregularly in 3D space. These points are stored in the order in which they are scanned, forming a unique trajectory according to the specific type of scanner [9]. However, this pattern can easily become irregular when the laser beam emitted by the scanner meets objects of sharply differing heights or the data undergoes processes such as merging, filtering, or segmentation. Much of ALS data processing relies on the operations of querying points at specific locations along with their neighbors. However, such operations cannot be efficiently executed when ALS point data are stored in common data structures such as the stack or queue [10]. A triangular irregular network (TIN) can be a solution for the operations, but the large computational overhead in forming a TIN with enormous amounts of ALS data is a drawback.

**Figure 1.**
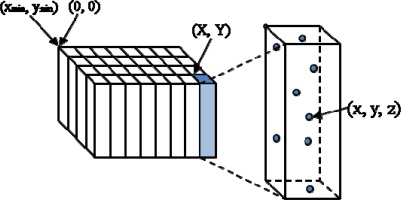
Virtual grid [10].

In order to rectify this situation, we propose to use a virtual grid [11] similar to the pseudo-grid introduced by Cho *et al.* [12] which previously has been adopted as a very effective data structure for ALS data processing. As shown in Figure 1, a 2D void array in C language, covering the entire geographic extent of the ALS data, is first generated. Each cell of the array points to the head of a dually linked list that stores point information such as 3D coordinates, intensity, and others. To place a point on the virtual grid, as shown in Equation 1, the planar (x, y) coordinates of the point are converted to shorter (X, Y) integers representing the cell coordinates of the virtual grid. Then, the point is attached to the linked list belonging to the cell (X, Y) of the virtual grid. To retrieve points near a specific location (x’, y’), the planar coordinates are converted to the cell coordinates of the virtual grid, and all points contained at the linked list belonging to the cell are accessible.

(1) $$\begin{array}{l} X=\mathrm{INT}(x-x_{\text{min}})/n_{\mathrm{cs}}\\ Y=\mathrm{INT}(y-y_{\text{min}})/n_{\mathrm{cs}}, \end{array}$$

where (x_min_*, y_min_*) are the minimum coordinates of the whole data, and *n_cs_* is the geometric size of a cell in the real coordinate system, which is equal to the target resolution of the resulting raster file in this study. The virtual grid is a memory-intensive structure, throughput being limited to some extent in that all of the data is stored in the main memory. However, this weakness can be overcome if, as in parallel systems, enough resources are provided.

### 2.2. Parallel processing and Performance Evaluation

Parallel processing is the concept of using multiple computers or processors to reduce the time needed to solve a heavy computational problem, operating on the principle that large problems can often be divided into smaller ones and then solved concurrently. A parallel processing system denotes a multiple-processor computer system consisting of centralized multiprocessors or multi-computers. For parallel processing, a parallel algorithm needs to be devised and its performance can be evaluated with reference, for example, to speedup and efficiency. If the algorithm is to handle a huge amount of data, load scalability or linearity should be considered. Detailed descriptions of the various aspects of parallel processing follow.

#### 2.2.1. Parallel Machines

A parallel processing system is called a centralized multiprocessor system if all processors share access to a global memory that supports communication and synchronization among processors. This system can be extended to super computers or massive parallel processing (MPP) computers if very many processors are integrated and each processor is provided with an individual memory connected with other processors by a bus. This kind of computer offers very high performance but requires a special operation system and incurs heavy construction costs in general.

Alternatively, a set of computers can be constructed as a parallel processing system, in other words a cluster system, if they are interconnected by a network. Recently, as microprocessors have become greatly enhanced and the needs for parallel computation have increased, relatively cheaper PC clusters have come available and have proved to be popular in general purposes [13–16]. Computers in a PC cluster are little different from ordinary personal computers or workstations, and the processor in each computer can interact with others by a message passing protocol such as MPI (Message Passing Interface) [17] or PVM (Parallel Virtual Machine) [18], through either an Ethernet or other higher-speed inter-connections. A general PC cluster consists of a master node, several slave nodes and network devices. A master node takes the role of the user interface, data input/output/distribution and control of slave nodes, and the slave nodes are responsible for data processing. In this study, we used a PC cluster to evaluate the proposed parallel algorithm.

#### 2.2.2. Performance Evaluation

##### (1) Speedup

The speedup *S_p_(n)* is defined as the ratio of the time required by an optimal sequential algorithm using one processor versus that required by a parallel algorithm, using *p* processors, processing input data of size *n* [18]. Ideally, *S_p_(n)* should be *p*, but does not attain *p*, owing to overhead such as communication between processors and other delays:

(1) $$S_{p}(n)=\frac{T(n)}{T_{p}(n)}$$

where *T(n)* is the time complexity of an optimal sequential algorithm and *T_p_(n)* is that of a parallel algorithm using *p* processors when the input data size is *n*.

##### (2) Efficiency

The efficiency *E_p_(n)* is defined as the ratio of the time required by a parallel algorithm using one processor versus that required by a parallel algorithm using *p* processors multiplied by the value *p* [18]. Theoretically *E_p_(n)* should be equal to 1, but normally, 1 cannot be attained:

(2) $$E_{p}(n)=\frac{T_{1}(n)}{{\mathrm{pT}}_{p}(n)}$$

##### (3) Load scalability and linearity

Another quality a parallel algorithm should have is load scalability. It is said that a system has load scalability if it has the ability to function gracefully without undue delay or resource consumption under light, moderate, or heavy loads [20]. In this context, more concrete measurement is linearity, which means an algorithm runs with linear time complexity (*O(n)*), that is, the running time increases linearly relatively to the size of the throughput. This quality is crucial in huge data processing contexts, because it has the decisive influence on the processing schedule and the corresponding throughput size.

### 2.3. IDW and Local Minimum Filtering

Interpolation is a method of constructing new date points with a limited number of known data points. Interpolation can be applicable to converting irregularly distributed data into a raster image, and a raster DSM can be made by interpolating the altitude information of ALS points. There are several methods applicable to interpolating ALS data, such as nearest neighbor, natural neighbor, Kriging, IDW, and others. Among them, IDW is easy to implement and is furnished in many GIS software, and thus it has been adapted to various applications in order to generate DSM [21–23]. The formula for IDW is shown in equation 4:

(3) $$\begin{array}{l} f(x)=\frac{\sum_{n=1}^{k}w(x_{n})\times f(x_{n})}{\sum_{n=1}^{k}w(x_{n})}\\ w(x_{n})=\frac{1}{d{\left(x,x_{n}\right)}^{p}} \end{array}$$

where, *f(x)* is the value at location *x*, *f(x_n_)* is the value at neighboring point *x_n_, w(x_n_)* is the weighting factor for point *x_n_, d(x, x_n_)* is the Euclidian distance between *x* and *x_n_*, and *p* is an exponential number equal to or greater than 2. The size of neighborhoods to be used in interpolation can be specified in terms of search radius, which was adopted for this paper, the number of points(*k*-nearest neighbor), or a combination of the two. Previous researchers have reported on the computational performance improvement of IDW using MIMD(Multiple Instruction Multiple Data) parallel computers. For example, Armstrong and Marciano [24] improved, by means of parallel processing, an IDW algorithm that uses brute-force search. In a succeeding paper, Armstrong and Marciano [25] tested a more efficient IDW algorithm based on local search [26]. An MPP system with thousands of processors based on SIMD (Single Instruction Multiple Data) architecture was used to improve Clarke’s model [27], and a quadtree approach to decompose the interpolation problem in Grid Computing environments was followed for load balancing [28]. The parallel algorithm developed in this study, using local search on SIMD architecture, is similar to that of Armstrong and Marciano [27]. However, presented in this paper are the means of producing corresponding result with sequential processing in a PC cluster system, which are described, in the next section, as solving boundary problem and interpolation center problem.

The local minimum filter is an operation that evaluates the value at a given location by endowing the smallest value in a window surrounding the location. As the window moves, relatively larger values than the surroundings are substituted by the locally smallest value in the instant window. The filter can be used to remove non-terrain objects that are higher than the surrounding terrain if the proper window size, slightly larger than the largest object in the scene, is set. Thus a DTM can be produced by applying the filter to a raster DSM [29].

There have been many studies on high-performance DSM or DTM generation from ALS data, from the viewpoint of accurate representation of sites. IDW and local minimum filtering were adopted in the present study because the computational overhead can be controlled by regulating parameters such as search radius and window size. Another reason is that they are algorithmically linear time operations assuming that retrieving data at a random location takes constant time. The constant time retrieving can be achieved by using a virtual grid. Thus, IDW and local minimum filtering were implemented to test two aspects of the efficiency of the proposed parallel algorithm: any advantage over a sequential algorithm in heavy-overhead processing, and any capability of dealing efficiently with large throughput, that is, load scalability.

## 3. Algorithm Development

### 3.1. Overall Algorithm

**Figure 2.**
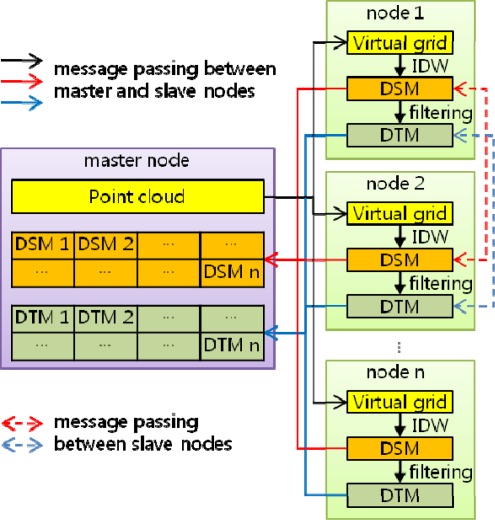
Data-flow diagram.

The first step was to distribute point data from the main node to the slave nodes by message passing. As the second step, the points transferred to each of the slave nodes were stored in the virtual grid and then IDW was applied to create a raster DSM. The third step was to create a DTM, by local minimum filtering, from the DSM. As the final step, the partial DSM and DTM in a raster format created by each slave node were transferred to the master node and two raster files of the DSM and DTM were built covering the whole area. The overall process is shown in Figure 2.

A massive data set can be loaded to random access memory (RAM) and easily accessed in parallel systems based on a large shared memory or distributed local memories which are connected with a bus. However, in a PC cluster system, data in the master node or a storage node are accessible in other nodes only after they are physically transferred through a network media by message passing. External network devices have been highly developed, but are still very slow contrary to data flows in RAM itself or through a bus. With an Ethernet connection, which is a typical network technology employed with a PC cluster system, frequent transfers of small data packet are inefficiency, because data is transferred in a unit which is made up of header, data and CRC(Cyclic Redundancy Check) taking at least 48 bytes for the data regardless of its size [30]. For the application, points were distributed to each node packed in blocks (e.g., 100,000 points/transfer) in the first step.

For optimal parallel processing performance, it is necessary to distribute an equal workload to each node, but that is not so easily achievable in ALS data processing due to the irregular distribution and density of points. Instead, the point cloud was equally divided geometrically and each apportionment was transferred to the slave nodes under the hypothesis that points will almost uniformly exist within each part if the target area is sufficiently large and surveyed under similar conditions. More delicate distributing methods will be considered in succeeding studies.

It is a basic assumption that the DSM and DTM values at the corresponding locations in sequential and parallel processing should be the same. However, the correspondence can be broken in two problematic situations: the boundary problem and the interpolation center problem. The former occurs when the data near the boundary of each node are processed without consideration of the data transferred to the neighboring nodes, and the latter arises when the coordinate origin is set without consideration of interpolation centers of neighboring node. More detailed descriptions of the situations and propositions follow in Sections 3.2 and 3.3, respectively.

### 3.2. Boundary Problem

**Figure 3.**
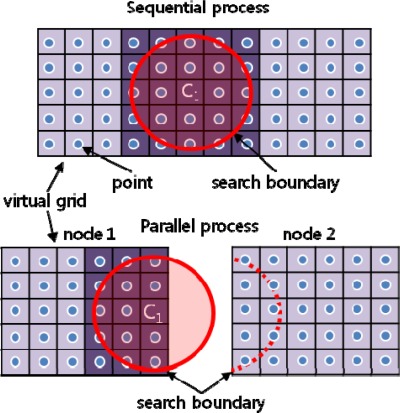
Different no. of searchable cells between sequential and parallel processing.

In sequential processing, any data within a given distance from a location of interest can be easily referenced to estimate the value of the location. However, in parallel processing, if the location is near the boundary of a node, not all of the data within the distance can be searched. As illustrated in Figure 3, in sequential processing, the value V1 is evaluated considering the points in 25 cells residing in search boundary, but in parallel processing, the number of referenced cells for V1 decreases to 15, necessarily resulting in a different interpolation value.

There are two possible solutions: one is to transfer the original data block between neighboring nodes, allowing data overlap, and the other is to transfer the partially processed data block without data overlap.

### Method 1: Transferring data block allowing overlap of original data

**Figure 4.**
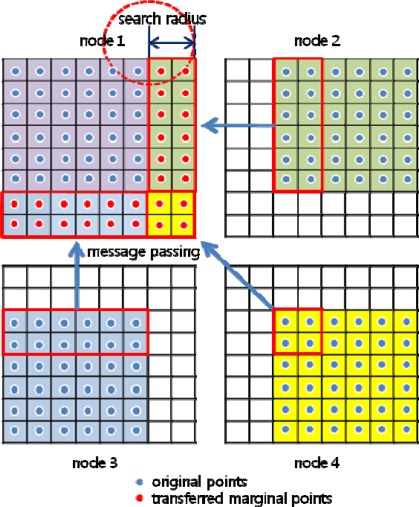
Transfer of marginal points.

**Figure 5.**
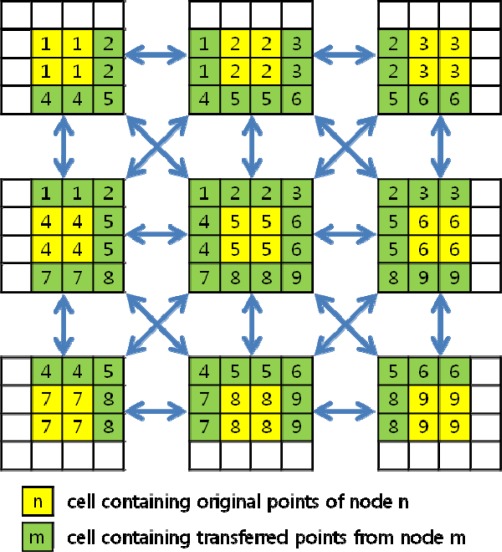
Transfer of marginal points among slave nodes.

Points to be searched over the boundary are packed in a block and transferred from neighboring nodes via message passing, as shown in Figure 4. Here and after, the idea of packing data in a block is to prevent the inefficiency stated in 3.1. The virtual grid is expanded according to the search radius, and the transferred marginal points are stored to the expanded cells. The transfer is done mutually between the two neighboring nodes. As illustrated in Figure 5, transmitting and receiving occurs concurrently, and a node (node 5 in this case) can transfer points in a maximum of 8 directions if it is fully enclosed by other nodes.

After the transfer is completed, interpolation is executed and filtering follows. A similar data transfer, applied to the interpolation process, is also employed to the filtering process. The difference is that the data type is not point in a virtual grid but digital value in the raster DSM produced through the interpolation process.

This method is straightforward but entails the disadvantage that each node is induced to have overlapped data, thus using more memory. Furthermore if more marginal points are needed, as when interpolating again with a larger search radius, the virtual grid should be wholly reallocated with the additionally transferred data or should be geometrically related to an additional virtual grid for storage of the additional data.

### Method 2: Transferring partially interpolated value block without overlap of original data

**Figure 6.**
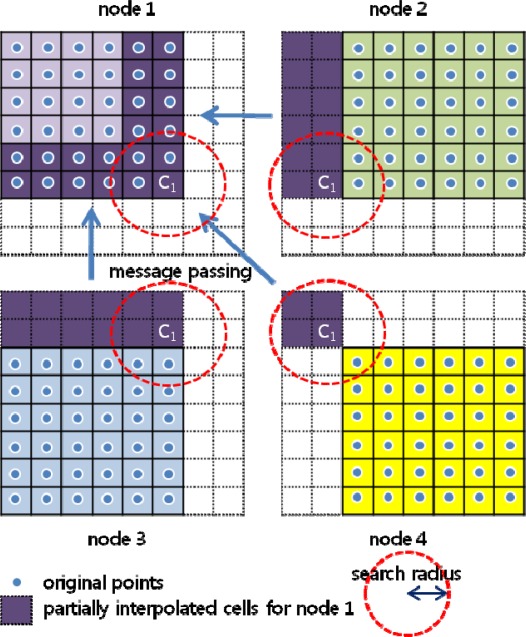
Transfer of intermediate vector.

In this method, instead of transferring original points, partially interpolated values at the corresponding locations in neighboring nodes are gathered in order to determine the final value of a given location. In other words, interpolation for a cell is executed in corresponding cells of neighboring nodes, after which the partially interpolated values are transferred to the original cell being integrated, to determine the completely interpolated value. In IDW, the interpolated value is evaluated as the ratio of ∑*w* and ∑*w•v* in equation 4, and each term can be modified to the form of $\sum_{N}\sum_{p\in N}w_{p}$ and $\sum_{N}\sum_{p\in N}w_{p}•v_{p}$ where p denotes the points within a given radius from the interpolation center of the given cell belonging to node N. In Figure 6, a cell C_1_ in node 1 has a corresponding cell in each neighboring node, the four cells having vector I_N_, defined as equation 5:

(4) $$I_{N}=\left(\sum_{p\in N}w_{p},\sum_{p\in N}w_{p}•v_{p}\right)$$

Where, N denotes the node number. I_N_ is evaluated in each node and transferred to node 1 via message passing. The final interpolated value I of the cell C_1_ is determined by equation 6.

(5) $$I=\sum_{N}\sum_{p\in N}w_{p}•v_{p}/\sum_{N}\sum_{p\in N}w_{p}$$

In filtering, F_N_, instead of I_N_, is defined as the partially filtered value in a given cell and in its corresponding cells in the virtual grid of each neighboring node. The F_N_ of each node is transferred to the given cell, and the final value determined is the minimum among the transferred values.

In both interpolation and filtering, virtual value transfer is done mutually between the two neighboring nodes, as shown in Figure 4. In method 2, a cell’s transferred data is a vector consisting of one or two variables of double precision float, whereas in method 1, the data consists of several 3D point coordinates because there can exist several points in a virtual grid cell. Furthermore, this method can cope with different searching radii without necessitating modifications to the original virtual grid. In these respects, we adopted method 2 for use in this study.

### 3.3 Interpolation Center Problem

**Figure 7.**
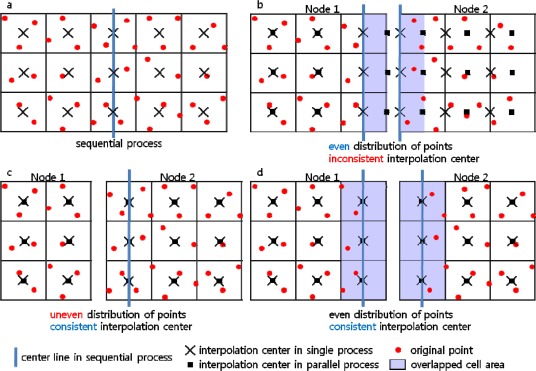
Selecting interpolation center.

The interpolation center of a cell in parallel processing, if the coordinate origin in a node is set without consideration of the neighboring nodes, will not necessarily be geometrically coincident with the corresponding one in sequential processing, resulting in a different interpolated-value. Figure 7a is a virtual grid, showing the original points and the interpolation center of each cell, in sequential processing. For parallelization, the point distribution and the scheme by which the interpolation center is chosen can be varied, as shown in Figure 7b, Figure 7c and Figure 7d. Detailed descriptions follow.

### Case A (Figure 7a)

The interpolation center is in the middle of each cell and the center line is in the horizontal center of the minimum bounding rectangle. The interpolation center, alternatively, can be in the corner or an arbitrary location, provided that it is in the same position in every cell.

### Case B (Figure 7b)

The points are divided by the geometric center line, which becomes the column origin of node 2, and distributed to the two nodes. In this case, the interpolation centers in node 2 are shifted, and thus they cannot be consistent with those in sequential processing. This will result in different interpolated values.

### Case C (Figure 7c)

The points are divided by a vertical line of cell boundaries near to the geometric center line and distributed, and the column origin of node 2 is set to the vertical line, which does not result in a shift of the interpolation centers in node 2. However, this schema has the weakness of geometrically uneven point distribution, which, if a different cell size is applied to the established virtual grid, requires additional point transfer between the two nodes.

### Case D (Figure 7d)

The points are divided by the geometric center line and distributed, and the column origin of node 2 is set to the left boundary of the right-end cells of node 1, in which case no shift of the interpolation centers in node 2 is imposed. In this case, both the right-end cells of node 1 and the left-end cells of node 2 have the same interpolated value, but one of them can easily be eliminated. Thus this schema was adopted for use in this study.

## 4. Implementation and Discussion

### 4.1. Test Data and System Configuration

The proposed algorithm was tested with real ALS data. The specifications of the data and of the parallel system are listed respectively in Tables 1 and 2, and the processing parameters are listed in Table 3.

The maximum 134 million points and their cropped datasets were processed and processing time was determined. The processing time has two main components, pure processing time and transfer time. Pure processing time includes: (1) reading and parsing data files; (2) interpolating and filtering; (3) writing final results on hard disk drives, all of which both the sequential process and the parallel process require. The adopted data files are in the TerraScan binary format, which includes a series of 3D coordinates, intensity, flight line information, and other parameters [31]. So the delay concerned with reading and parsing the files (up to 5GB for dataset 6) to extract 3D coordinate for each point is not negligible. The transfer time is the Ethernet networking time used (1) to transfer point data from the master node to slave nodes; (2) to exchange partially interpolated and filtered values among nodes; and (3) to transfer final results from the slave nodes to the master node. Speed up and efficiency were confirmed for dataset 3 and dataset 4, because the larger data size brought about a system halt in sequential processing on a single node. Load scalability was confirmed from dataset 1 through to dataset 6 with 8 slave nodes.

### 4.2. Performance Evaluation and Discussion

**Figure 8.**
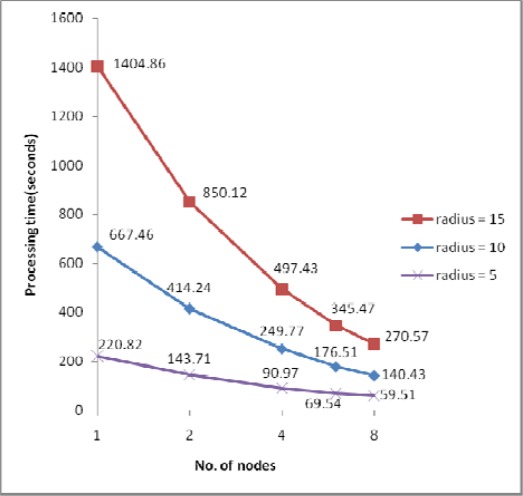
Processing time for dataset 3.

The processing times along with the speedups and efficiencies for dataset 3 (17.9 million points) with three different IDW searching radii (5m, 10m and 15m) are presented in Figures 8, 9a and 9b. In Figure 8, 1 node denotes sequential processing in which only a single processor was used with a sequential algorithm, whereas 2, 4, 6 and 8 nodes denote parallel processing with the indicated number of processors. The figure shows performance for the proposed parallel processing method. In Figure 9a and Figure 9b, it is clear that speedup and efficiency increase both with increasing search radius and according to the number of processors with respect to the test set. Speedup increased by 40%, from 3.71 to 5.19, and efficiency by 41%, from 0.46 to 0.65, as the size of the search radius was increased from 5m to 15m when 8 nodes were used. By contrast, speedup increased by only 16%, from 2.43 to 2.82, and efficiency by only 16%, from 0.61 to 0.71, when 4 nodes were used. The results confirm two expectations: (1) as the search radius becomes larger, the transfer time and the file manipulation time do not increase significantly compared with the computation time (here, “computation time” denotes only the interpolation and filtering processing times, which apply in both sequential and parallel processing); (2) as the number of nodes becomes larger, the computation time decreases almost in exact proportion to the number of nodes, whereas the transfer time and the file management time are not significantly increased. Therefore, the proposed parallel algorithm is more favorable in the case of (1) a larger search radius, that is, a heavier computational load, (2) a system with more processors. This finding can also be applicable to the varying filter size.

**Figure 10.**
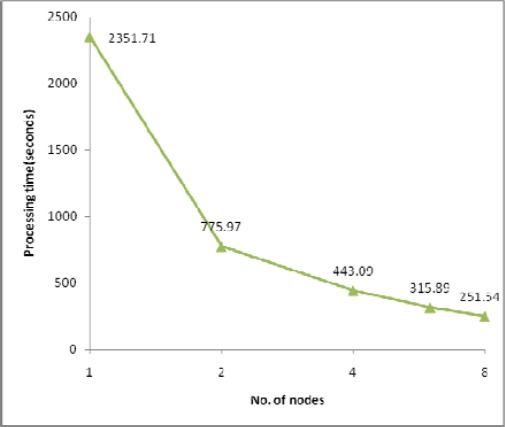
Processing time for dataset 4.

The processing times along with the speedups and efficiencies for dataset 4 (31.7 million points) with one IDW search radius (10m) are presented in Figures 10, 11a and 11b. As shown in Figure 10 and Figure 11a, sequential processing took 2351.71 seconds, and 2 node parallel processing took 775.97 seconds; that is, the speedup according to Equation 2 was 3.03, whereas the speedup under the same conditions was 1.61 in the case of dataset 3. This result implies that, in the case of dataset 4, sequential processing should use page memory for processing an abruptly larger input, and that parallel processing can yet run using only RAM (Random Access Memory). Page memory is a virtual memory that relies on a hard disk drive when the system is given a task larger than can be handled by physical RAM, which necessarily slows down the overall processing speed. Thus, as shown in Figure 11b, parallel processing achieves a speedup and efficiency better than 1 for each test set, which is called superlinearity. This implies that PC cluster processing can be a solution to the problem of processing large ALS datasets that cannot be efficiently processed by a sequential system having limited computational resources.

**Figure 12.**
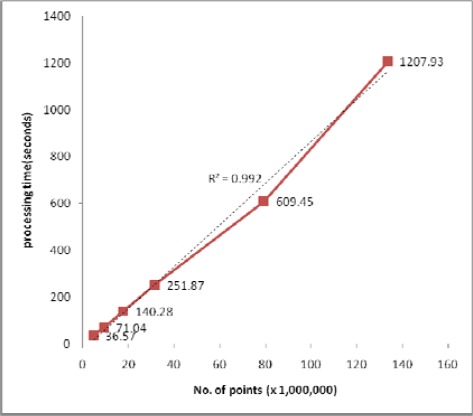
Processing times for 8 node PC cluster.

An additional test to determine load scalability was conducted and the results plotted in Figure 12 show that the processing time increased almost in exact proportion to the size of the input data; in other words, the algorithm was shown to be a linear time operation. The performance of linear operation is expected to be maintained until at least one slave node suffers performance declination when the size of the data is so large as to require page memory.

**Figure 13.**
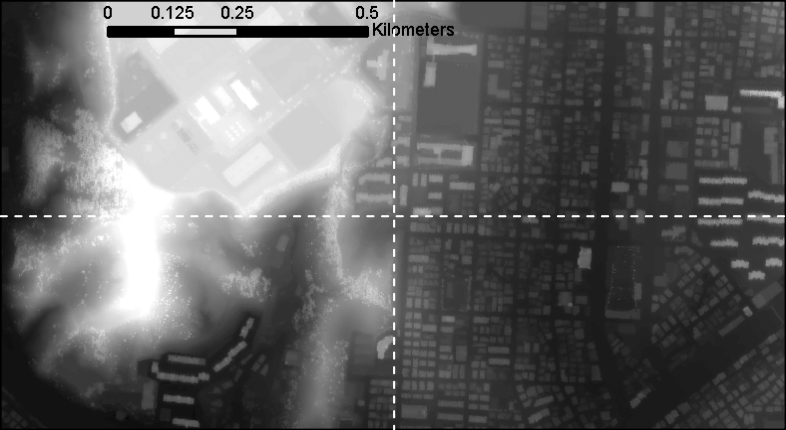
Raster DSM produced by node 1 through to node 4 from dataset 1.

Figure 13 shows the resultant DSM of dataset 1, the white dashed line on the image illustrating the border of the nodes when processed with 4 slave nodes. In contrast to the result for sequential processing, the difference between the corresponding DSM and DTM pixels was ±0.0, measured in single precision float accuracy.

**Figure 14.**
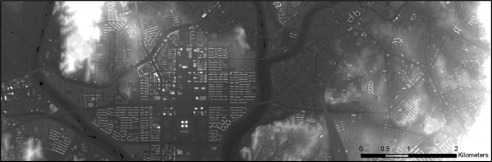
Raster DSM produced from dataset 6.

**Figure 15.**
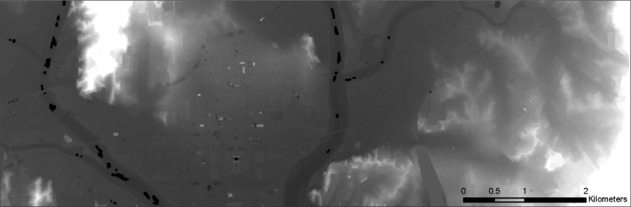
DTM filtered from DSM produced from dataset 6.

Finally, the DSM and DTM for dataset 6, covering about 36 km^2^, are shown respectively in Figures 14 and 15. Figure 15 shows some still-existing buildings, which could be excluded by employing a larger filter size, but at the cost of some geometric accuracy.

## 5. Conclusions

This paper proposed a parallel processing method for DSM and DTM generation using a PC cluster and a virtual grid, as a methodology of efficient processing of huge amounts of ALS data. A raster DSM was generated from raw ALS point data by interpolating with inverse distance weighting (IDW), and a raster DTM was produced from the DSM by local minimum filtering. A methodology of dealing with boundary data and of selecting interpolation centers in the parallel processing was designed to ensure the same outcomes from the sequential processing. For performance assessment, real ALS data amounting to as many as 134 million points, and a PC cluster consisting of a master node and 8 slave nodes, were employed. Speedup, efficiency and linearity were determined in evaluating the proposed algorithm. The results showed that the parallel processing method can offer better speedup and efficiency when larger computational overheads were assigned and a system with more processors was used. Also, unexpectedly high speedup and efficiency were achieved when processing 31.7 million points and the maximum 134 million points with the proposed system. The computational experiments proved that parallel processing can be a solution to the problem of processing huge amounts of ALS data. The appropriateness of adopting virtual grid for the manipulation of ALS data processing with parallel processing was verified by the result that the proposed algorithm functioned as a linear time operation. Moreover, the products from the proposed algorithm were completely identical to those of sequential processing.

The authors have discussed only DSM and DTM generation in a parallel processing environment. There are a number of complex filtering, segmentation and feature extraction algorithms for ALS data processing. Managing and displaying technologies for ALS data is also of importance. Most of the algorithms and operations are expected to be improved from the performance perspective, when parallel processing along with a virtual grid is used. The authors currently are developing a full-fledged ALS data processing system for the given PC cluster, which would be expected to be recognized as an advanced ALS data processing system. Notwithstanding, other high-performance computing technologies such as SMP (Symmetric Multiprocessors) and GPGPU (General Purpose Graphic Processing Unit) should also be considered in efforts to improve the performance of future ALS data processing.

## Figures and Tables

**Figure 9a:**
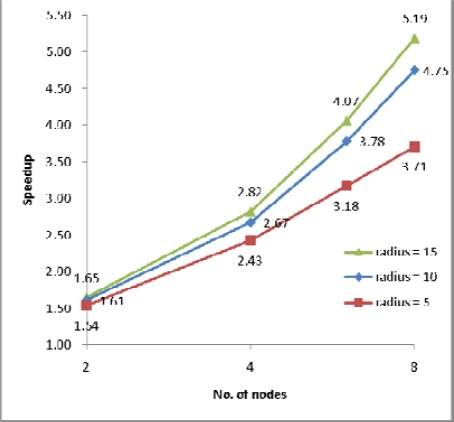
Speedup for dataset 3.

**Figure 9b:**
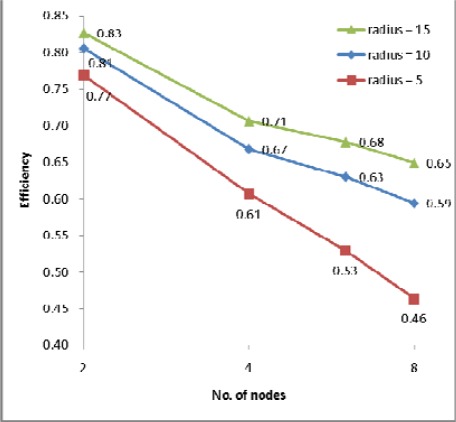
Efficiency for dataset 3.

**Figure 11a:**
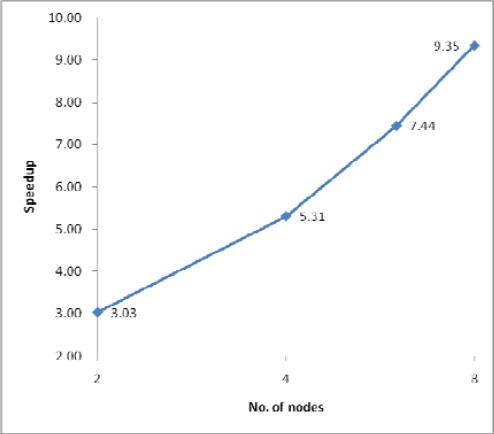
Speedup for dataset 4.

**Figure 11b:**
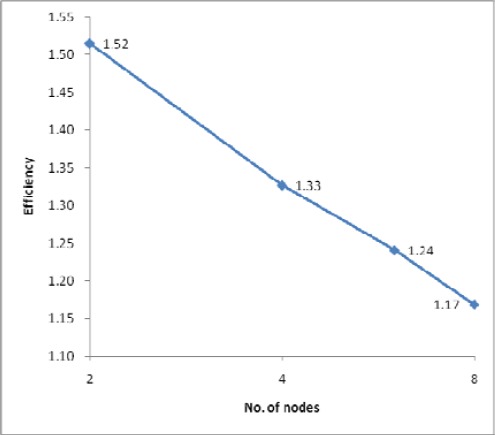
Efficiency for dataset 4.

**Table 1.** ALS data specifications.

Laser scanner	ALS ALTM 3070 system (Optech, Inc.)	
Target area	Daejeon, South Korea	
Preprocessing	Systematic error correction was applied.	Strip adjustment and blunder removal were not applied.
Dataset 1	4.8 × 10^6^ points covering1.5 × 0.8 km^2^ (3.7 points/m^2^)	cropped from dataset 6
Dataset 2	9.4 × 10^6^ points covering 3.0 × 0.8 km^2^ (3.7 points/ m^2^)	cropped from dataset 6
Dataset 3	17.9 × 10^6^ points covering 6.1 × 0.8 km^2^ (3.5 points/ m^2^)	cropped from dataset 6
Dataset 4	31.7 × 10^6^ points covering 6.1 × 1.7 km^2^ (3.1 points/ m^2^)	cropped from dataset 6
Dataset 5	79.3 × 10^6^ points covering 10.7 × 1.7 km^2^ (4.4 points/ m^2^)	cropped from dataset 6
Dataset 6	133.7 × 10^6^ points covering 10.7 × 3.4 km^2^ (3.7 points/ m^2^)	full dataset

**Table 2.** PC cluster specifications.

System configuration	Parallel Sequential	1 master node with 1, 2, 4, 6, 8 slave nodes 1 node

	Single Pentium 4 3.0 GHz, 1 GB RAM for each node	

Network	1Gb Ethernet	
Operating system	Windows XP sp3	
MPI library	MPICH 2.0 (following MPI 2.0 standard)	
Coding language	C++	

**Table 3.** Experimental parameters.

Virtual grid cell size	1m by 1m
IDW search radius	15m / 10m / 5m (for dataset 3), 10m (for other datasets)
IDW power	2
Filter size	30m by 30m

## References

[1] Flood M. (1999). Commercial Development of Airborne Laser Altimetry. Int. Arch. Photogramm. Remote Sens.

[2] Baltsavias E.P. (1999). A comparison between photogrammetry and laser scanning. ISPRS J. Photogramm. Remote Sens.

[3] Shan J., Sampath A. (2005). Urban DEM Generation from Raw LiDAR Data : a Labeling Algorithm and its Performance. Photogramm. Eng. Remote Sens.

[4] Han S.H., Lee J.H., Yu K.Y. (2007). An Approach for Segmentation of Airborne Laser Point Clouds Utilizing Scan-Line Characteristics. ETRI J.

[5] Healey R., Dowers S., Gittings B., Mineter M.J. (1997). Parallel Processing Algorithms for GIS.

[6] Clematis A., Mineter M., Marciano R. (2003). High performance computing with geographical data. Parallel Comput.

[7] Yang C., Hung C. Parallel Computing in Remote Sensing Data Processing.

[8] Plaza A.J., Chang C. (2007). High Performance Computing in Remote Sensing.

[9] Wehr A., Lohr U. (1999). Airborne laser scanning - an introduction and overview. ISPRS J. Photogramm. Remote Sens.

[10] Cormen T.H., Leiserson C.E., Rivest R.L., Stein C. (2001). Introduction to Algorithms.

[11] Han S.H. (2008). Efficient segmentation of ALS point cloud utilizing scan line characteristic.

[12] Cho W., Jwa Y.S., Chang H.J., Lee S.H. (2004). Pseudo-grid Based Building Extraction Using Airborne Lidar Data. Int. Arch. Photogramm. Remote Sens.

[13] Quinn M.J. (2004). Parallel programming in C with MPI and OpenMP.

[14] Bader D.A., Pennington R. (2001). Cluster computing: Applications. Int. J. High Perform. Comput.

[15] Almeida F., Gomez J.A., Badia J.M. Performance analysis for clusters of symmetric multiprocessors.

[16] Top 500 supercomputer sites.

[17] Message Passing Interface.

[18] Parallel Virtual Machine.

[19] JaJa J. (1992). An Introduction to parallel algorithms.

[20] Bondi A.B. Characteristics of Scalability and Their Impact on Performance.

[21] Bartier P., Keller C.P. (1996). Multivariate interpolation to incorporate thematic surface data using inverse distance weighting(IDW). Comput. Geosci.

[22] García-León J., Felicísimo A.M., Martínez J.J. (1999). A methodological proposal for improvement of digital surface models generated by automatic stereo matching of convergent image networks. Int. Arch. Photogramm. Remote Sens.

[23] Gonçalves G. Analysis of interpolation errors in urban digital surface models created from LIDAR data.

[24] Armstrong M.P., Marciano R. (1994). Inverse-Distance-Weighted Spatial Interpolation Using Parallel Supercompters. Photogramm. Eng. Remote Sens.

[25] Armstrong M.P., Marciano R. (1996). Local Interpolation Using a Distributed Parallel supercomputer. Int. J. Geogr. Inf. Syst.

[26] Clarke K. C. (1990). Analytical and Computer Cartography.

[27] Armstrong M.P., Marciano R. (1997). Massively Parallel Strategies for Local Spatial Interpolation. Comput. Geosci.

[28] Wang S., Armstrong M.P. (2003). A Quadtree Approach to Domain Decomposition for Spatial Interpolation in Grid Computing Environments. Parallel Comput.

[29] Rowland C.S., Balzter H. (2007). Data Fusion for Reconstruction of a DTM, Under a Woodland Canopy, From Airborne L-band InSAR. IEEE Trans. geosci. remote sens.

[30] Comer D.E. (2006). Internetworking with TCP/IP Vol. 1.

[31] TerraSolid Ltd. Homepage.

